# Safety and Efficacy of Canakinumab for the Prevention and Control of Type 2 Diabetes Mellitus and Its Complications: A Systematic Review

**DOI:** 10.7759/cureus.67065

**Published:** 2024-08-17

**Authors:** Nidhi Lanka, Prakash Acharya, Shikha Virani, Sumayya Afreen, Arvin Perthiani, Elizabeth Sangster, Ana P Arcia Franchini

**Affiliations:** 1 Internal Medicine, California Institute of Behavioral Neurosciences & Psychology, Fairfield, USA; 2 Obstetrics and Gynaecology, California Institute of Behavioral Neurosciences & Psychology, Fairfield, USA; 3 General Surgery, California Institute of Behavioral Neurosciences & Psychology, Fairfield, USA; 4 Psychiatry and Behavioral Sciences, California Institute of Behavioral Neurosciences & Psychology, Fairfield, USA; 5 Research, California Institute of Behavioral Neurosciences & Psychology, Fairfield, USA

**Keywords:** major adverse cardiac events (mace), impaired glucose tolerance, glycated hemoglobin (hba1c), interleukin 1β, canakinumab, type 2 diabetes mellitus (dm)

## Abstract

Today, diabetes mellitus (DM) is one of the leading causes of morbidity and mortality globally.In this grim context, while our current armamentarium of anti-diabetic agents is vast and increasingly available, glycemic control in a significant proportion of these patients continues to remain sub-optimal.This necessitates the exploration of other potential cellular pathways and targets to effectively manage this notorious disease and its numerous complications. Inflammatory responses are thought to be implicated in the decline of pancreatic beta-cell function, with interleukin-1 beta (IL-1β) playing an important role in these pathways. Canakinumab, a human monoclonal anti-IL-1β antibody, operates by reducing inflammation, potentially safeguarding or enhancing pancreatic beta-cell function. This systematic review aims to study the safety and efficacy of canakinumab in the prevention and control of type 2 diabetes mellitus (T2DM) and its complications. This study was conducted in accordance with the PRISMA 2020 Guidelines. PubMed including MEDLINE, Google Scholar and Cochrane Library were used as information sources and randomized clinical trials and retrospective observational studies evaluating patients with T2DM or impaired glucose tolerance with/without complications receiving canakinumab, compared with placebo or standard therapy and reporting about glycemic indicators including hemoglobin A1C (HbA1C) or blood sugar levels (BSL) or insulin levels and/or inflammatory indicators including high-sensitivity C-reactive protein (hsCRP) or interleukin-6 (IL-6) were included. Non-randomized clinical trials, animal studies, review articles, case reports, case series, studies not in the English language and those evaluating type 1 DM were excluded. In total, 271 studies were identified to be included in this study. Subsequently, 27 were found to be duplicate records and were eliminated. Manual screening of title/abstract of 244 records was done which found 207 to be ineligible and 37 studies were shortlisted. These were retrieved and full-text screening was undertaken which resulted in the exclusion of 28 reports due to the following reasons: ineligible study design (17), studies evaluating type 1 DM (three), studies evaluating anakinra (one), trial being canceled (three) and duplicate studies (four). Subsequently, a total of nine studies were included in the final review. All studies were included post quality appraisal. We found that canakinumab had a modest but mostly non-significant effect on glycemic parameters including HbA1C, while having a consistently significant reduction in systemic inflammatory parameters like hsCRP and IL-6. Additionally, it was found to have a significant reduction in incident major adverse cardiovascular events (MACE). Canakinumab was also found to be safe and well-tolerated in all patient populations. Although canakinumab did not reduce incident T2DM, an exploration of alternative pathways and targets implicated in the pathogenesis of this disease process is warranted for the prevention and control of T2DM.

## Introduction and background

Today, diabetes mellitus (DM) is one of the leading causes of morbidity and mortality globally, affecting over 529 million people worldwide, with this number projected to cross 1.31 billion by 2050 [1]. Within this grim context, while our current armamentarium of anti-diabetic agents is vast and increasingly available, glycemic control in a significant proportion of these patients continues to remain sub-optimal [2]. This necessitates the exploration of other potential cellular pathways and targets to effectively manage this notorious disease and its numerous complications.

Subclinical inflammation has long been considered to be involved in the pathogenesis of insulin resistance and Type 2 DM (T2DM). Several studies indicate that inflammatory responses are implicated in the decline of pancreatic beta-cell function, with preclinical evidence emphasizing the role of interleukin-1 beta (IL-1β) in these pathways [3-6]. Additionally, it has been found that acute-phase reactants such as high-sensitivity CRP (hsCRP) and pro-inflammatory cytokines like IL-1β and IL-6 are increased in T2DM [7]. It is also of note that pancreatic beta-cells exhibit the highest density of IL-1β receptors among body tissues. Thus, IL-1β seems to be a reasonable target for T2DM control that warrants further study. Subsequently, preclinical and clinical studies have shown that inhibiting IL-1β activity, either through a neutralizing IL-1β antibody like canakinumab or an IL-1 receptor antagonist like anakinra can enhance pancreatic beta-cell secretory function and reduce HbA1C levels in T2DM patients [8-10].

Canakinumab is a human monoclonal anti-IL-1β antibody, approved for use in cryopyrin-associated periodic syndromes (CAPS) (an IL-1β driven inflammatory disease) and under investigation for use in rheumatoid arthritis, systemic juvenile idiopathic arthritis and refractory gout. It first came to the fore in cardiometabolic disease control via the Anti-inflammatory Therapy with Canakinumab for Atherosclerotic Disease or CANTOS trial in 2017 which found that 150 mg of canakinumab significantly reduced rates of major adverse cardiovascular events (MACE) in 10,061 patients with previous myocardial infarction (MI) and an hsCRP≥ 2mg/L [11]. This landmark trial prompted its consideration in other disease processes which share similar causative inflammatory pathways. In fact, it is the CANTOS trial and the ‘common soil’ hypothesis for T2DM and cardiovascular disease that suggested a shared precursor for these two distinct yet overlapping entities over two decades ago that served as an inspiration in undertaking this review [12].

This systematic review aims to study the safety and efficacy of canakinumab, an IL-1β antibody in the prevention and control of T2DM and its complications.

## Review

Methods

This study was conducted in accordance with the Preferred Reporting Items for Systematic Reviews and Meta-analyses (PRISMA) 2020 Guidelines [13].

Search Strategy 

PubMed including MEDLINE, Google Scholar and Cochrane Library were used as information sources with the last search being performed on 05/04/2023 at 00:23. Relevant keywords combined with Boolean operators were used, with advanced search options employed where appropriate. The keywords used for PubMed were ‘(diabetes OR hyperglycemia OR prediabetes OR insulin resistance OR glucose intolerance) AND (canakinumab OR interleukin-1 beta antagonist)’ in combination with Medical Subject Headings (MeSH) terms. Filters applied were English language studies published in the last 10 years with free full text available. The search strategy used for Google Scholar was ‘diabetes AND canakinumab, diabetes OR hyperglycemia OR prediabetes OR insulin resistance OR glucose intolerance AND canakinumab OR interleukin-1 beta antagonist’ with ‘all in title, with at least one of the words, 2013-2023’ filters applied. The keywords used for the Cochrane Library were ‘Diabetes AND Canakinumab’ with filters for studies published in ‘2013-2023’ applied. The detailed search strategies with filters used and total number of results for each database can be viewed in Table 1 below.

**Table 1:** Search Strategy

Sr. No	Database	Search Strategy	Filters Applied	No. of Results
1	PubMed	(diabetes OR hyperglycemia OR prediabetes OR insulin resistance OR glucose intolerance OR ((((( "Diabetes Mellitus/blood"[Mesh] OR "Diabetes Mellitus/drug therapy"[Mesh] OR "Diabetes Mellitus/prevention and control"[Mesh] OR "Diabetes Mellitus/therapy"[Mesh] )) OR ( "Hyperglycemia/blood"[Mesh] OR "Hyperglycemia/drug therapy"[Mesh] OR "Hyperglycemia/prevention and control"[Mesh] OR "Hyperglycemia/therapy"[Mesh] )) OR ( "Prediabetic State/blood"[Mesh] OR "Prediabetic State/drug therapy"[Mesh] OR "Prediabetic State/prevention and control"[Mesh] OR "Prediabetic State/therapy"[Mesh] )) OR "Insulin Resistance"[Mesh]) OR ( "Glucose Intolerance/blood"[Mesh] OR "Glucose Intolerance/drug therapy"[Mesh] OR "Glucose Intolerance/prevention and control"[Mesh] OR "Glucose Intolerance/therapy"[Mesh] )) AND (canakinumab OR interleukin-1 beta antagonist OR ("canakinumab" [Supplementary Concept]) OR "Interleukin-1beta/antagonists and inhibitors"[Mesh])	Free full text, published in the last 10 years	216
2	Google Scholar	diabetes AND canakinumab, diabetes OR hyperglycemia OR prediabetes OR insulin resistance OR glucose intolerance AND canakinumab OR interleukin-1 beta antagonist	all in title, with at least one of the words, 2013-2023	6
3	Cochrane Library	Diabetes AND Canakinumab	2013-2023	49

Data Screening 

The studies retrieved were screened separately by two independent reviewers with conflicts being resolved by a third reviewer, as per pre-defined inclusion and exclusion criteria.

Inclusion criteria: Randomized clinical trials (RCTs) and retrospective observational studies evaluating patients with T2DM or impaired glucose tolerance (IGT) with/without complications receiving canakinumab, compared with placebo or standard therapy and reporting about glycemic indicators including HbA1C or blood sugar levels (BSL) or insulin levels and/or inflammatory indicators including hsCRP or interleukin-6 (IL-6) were included.

Exclusion criteria: Non-randomized clinical trials, animal studies, review articles, case reports and case series, studies not in the English language and studies evaluating Type 1 DM were excluded.

The studies identified after the initial search were first screened to delete duplicates. Next, a manual screening of the titles/abstracts was done and studies found to be ineligible were removed. Subsequently, manual screening of the full texts was done and the selected studies were further evaluated with quality assessment tools. In cases where full texts were not available, abstracts were considered.

Quality Appraisal

Two authors independently performed the quality appraisal with conflicts being resolved by a third author. Appropriate quality assessment tools were used for each study type to evaluate the risk of bias and quality of individual studies. The Cochrane Risk of Bias Assessment Tool was used for Randomized Clinical Trials, those having high risk or unclear risk of bias were removed. The Consolidated Standards of Reporting Trials (CONSORT) checklist extension for pilot and feasibility trials for pilot studies was used for pilot studies. Studies with a score of 70% or more were considered for final review.

Data Extraction and Synthesis

The following information was extracted in Microsoft Excel (Microsoft, Redmond, WA, USA) sheets from the eligible studies.

Baseline characteristics:* *Study name, author, year of publication, study design, sample size, age (median age, inter-quartile range), gender (% female), glycemic status (IGT/T2DM with duration), comorbidities, canakinumab dose and route, control group (placebo/standard therapy) and follow-up time.

Study outcomes: Changes in glycemic indicators including HbA1C, BSL (fasting/post-prandial glucose levels/oral glucose tolerance test (OGTT)), insulin levels (insulin secretion rate (ISR)/ HOMA-Insulin); inflammatory indicators including hsCRP, IL-6; indicators of diabetic complications including MACE, indicators of diabetic retinopathy, nephropathy; overall morbidity (hospital admissions) and mortality.

The above data was qualitatively synthesized.

Results

Study Selection 

**Figure 1:**
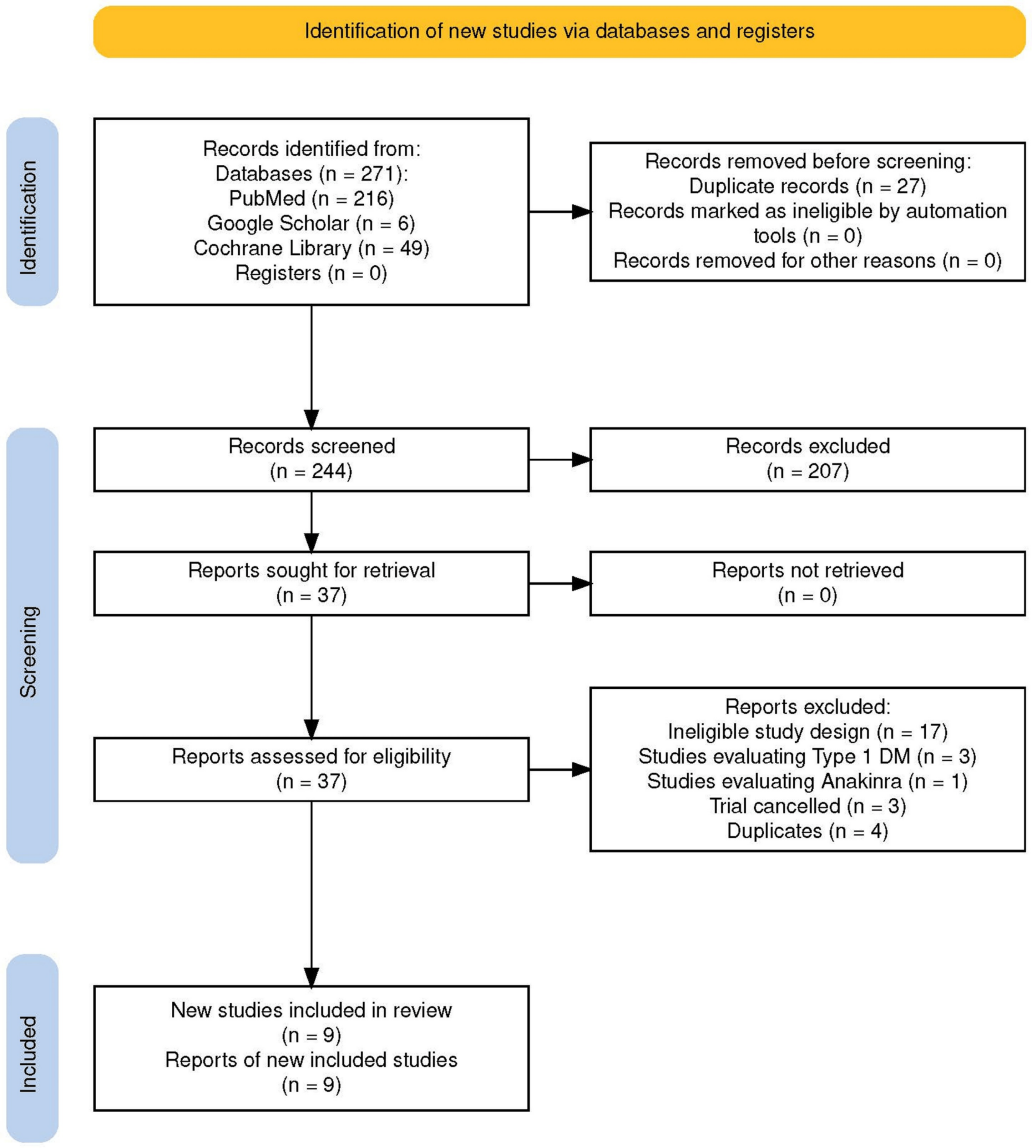
Preferred Reporting Items for Systematic Reviews and Meta-Analyses (PRISMA) flowchart depicting study selection and screening Flowchart generated using PRISMA 2020 software [14].

In total, 271 studies were identified to be included in this review, as per the mentioned search strategy (PubMed - 216, Google Scholar - 6, Cochrane Library - 49). Subsequently, 27 were found to be duplicate records and were eliminated before screening. Manual screening of title/abstract as per the pre-defined inclusion and exclusion criteria of 244 records was done which found 207 to be ineligible and 37 studies were shortlisted. These were retrieved and full-text screening was undertaken which resulted in the exclusion of 28 reports due to the following reasons - ineligible study design (17), studies evaluating Type 1 DM (3), studies evaluating anakinra (1), trial being canceled (3) and duplicate studies (4). Subsequently, a total of nine studies were included in the final review, as shown in Figure 1 below [14]. 

Study Characteristics

Baseline characteristics: A total of nine studies were included of which eight were RCTs and one was a pilot study. All studies were conducted between 2012-2022 at multiple locations with the majority being conducted in North America and Europe. A total population of 14,733 was studied with a mean age of 52.08 years (with mean age unspecified in one study) and a mean gender ratio of 33% females (with % female unspecified in two studies). All patients had a pre-existing diagnosis of pre-diabetes or diabetes with comorbidities like coronary artery disease (CAD), nephropathy, retinopathy and neuropathy mentioned where applicable. Various doses of canakinumab were administered either via the subcutaneous, intravenous or both routes and compared with placebo in all studies except the pilot study which did not have a control group. The follow-up period varied across studies. The baseline characteristics of all studies are summarized in Table 2 below.

**Table 2:** Baseline Characteristics Abbreviations: IGT- Impaired Glucose Tolerance, DM- Diabetes Mellitus, RCT- Randomized Clinical Trial, CAD- Coronary Artery Disease, PAD- Peripheral Artery Disease, IV- intravenous, sc- subcutaneous

Authors	Year	Location	Study Design	Sample Size	Mean Age (years), Range	Gender Ratio (% Females)	IGT or DM (no. of patients), Time since diagnosis (years)	Comorbidities	Canakinumab Dose and Route	Control	Follow-up Period
Hepprich M, et al. [15]	2022	Switzerland	RCT	116	71 (62-78)	31.6	DM (9), 3-16	Covid-19 (116), Obesity (116), Retinopathy (10), Nephropathy (25), Neuropathy (16), Stroke (5), CAD (25), PAD (4), Others (5)	450-750 mg, single IV dose	Placebo	3 months
Everett BM, et al. [8]	2018	Multiple locations	RCT	10,061	61+/-10	25.9	IGT (4960), DM (4057)	CAD (10, 061)	50, 150, 300 mg sc every 3 months	Placebo	3.7 years
Choudhury RP, et al. [16]	2016	Multiple locations	RCT	189	61.7+/-7.8	14	IGT (25), DM (162)	CAD (189)	150 mg sc monthly	Placebo	1 year
Stahel M, et al. [17]	2016	Switzerland	Pilot Study	6	66.2	16.6	DM (6)	Proliferative Diabetic Retinopathy	150 mg sc every 8 weeks x3	No control	24 weeks
Noe A, et al. [9]	2014	Multiple locations	RCT	231	54.4 (37.3-60.1)	53.8	DM (231), at least 6 months	No significant complications	0.03, 0.1, 0.3, 1.5, 10 mg/kg single IV dose	Placebo	24 weeks
Howard C, et al. [18]	2014	New Jersey, USA	RCT	1026	55.2+/-9.78	45.5	IGT (54), DM (972), 5.5+/-5.59 years	Retinopathy (22), Neuropathy (82), Nephropathy (21)	Low dose (0.03 mg/kg i.v. once), intermediate doses (0.1 and 0.3 mg/kg i.v. once, 5 and 15 mg s.c. monthly), medium doses (1.5 mg/kg i.v. once, 50 mg s.c. monthly and 150 mg s.c. once) and high doses (10 mg/kg i.v. once and 150 mg s.c. monthly)	Placebo	5.07+/-2.885 months
Hensen J, et al. [19]	2013	Germany	RCT	551	54.1	Non-specified	DM (551)	Non-specified	5, 15, 50, or 150 mg sc monthly x4	Placebo	4 months
Ridker PM, et al. [20]	2012	Boston, USA	RCT	556	54.1	43.6	DM (556), 3.7 years	Non-specified	5, 15, 50, 150 mg sc monthly x4	Placebo	4 months
Rissanen A, et al. [21]	2012	Finland	RCT	190	Non-specified	Non-specified	IGT (54), DM (136)	Non-specified	Non-specified	Placebo	4 weeks

Study outcomes: The data items for each study are summarized in the table below. Indicators of glycemic control such as HbA1C or BSL/OGTT are included for all studies. Insulin secretion indices such as ISR or HOMA-insulin resistance are also reported where available. Indicators of inflammation such as hsCRP and IL-6 levels are also included for all studies. Data about DM-specific complications such as CAD measured via MACE, N-terminal pro b-type natriuretic peptide (NT-proBNP), nephropathy via eGFR, retinopathy and overall complications including adverse drug reactions relating to canakinumab and 30-day mortality are also tabulated where available (Table 3).

**Table 3:** Study Outcomes Abbreviations: hsCRP- high-sensitivity C-reactive protein, BSL- Blood Sugar Level, OGTT- Oral Glucose Tolerance Test, ISR- Insulin Secretion Rate, MACE- Major Adverse Cardiovascular Events, NT-proBNP- N-terminal pro b-type natriuretic peptide

Authors	hsCRP	IL-6	HbA1C	BSL/OGTT	ISR	Diabetic Complications (MACE/Nephropathy/Retinopathy indicators)	30-day Mortality	Adverse Drug Reactions
Hepprich M, et al. [15]	Treatment effect (95%CI) (p-value) - 0·47 (0·27, 0·82) (p=0·009), 0·50 (0·27, 0·92) (p=0·027)	0·28 (0·11, 0·68) (p=0·005)	0·98 (0·93, 1·03) (p=0·431), 1·00 (0·94, 1·06) (p=0·926)	0·88 (0·74, 1·06) (p=0·170)	2·21 (1·09, 4·48) (p=0·029)	No significant differences in NT-proBNP, eGFR	Death-0·54 (0·13, 1·90) (p=0·347)	No significant differences
Everett BM, et al. [8]	35.3% reduction from baseline vs placebo, p<0.001	34.4% reduction vs placebo, p<0.001	Significant difference in 6-9 months, attenuated by 48 months	No consistent effect	Non-specified	MACE- Pre-DM - 0.86 (95% CI: 0.73 to 1.01), DM - 0.90 (95% CI: 0.77 to 1.05)	All cause mortality - 0.94 (0.83-1.06) (0.31)	Higher rates of neutropenia, fatal infection
Choudhury RP, et al. [16]	GMR: 0.56; 95% CI: 0.414 to 0.758; p = 0.0002	GMR: 0.580; 95% CI: 0.483 to 0.697; p < 0.0001	0.008 (–0.031 to 0.047), p = 0.68	0.068 (–0.017 to 0.154), p = 0.12	No significant difference in HOMA-insulin resistance	MACE- No significant difference	Non-specified	No significant differences
Stahel M, et al. [17]	0.01 point decrease, p=1.00	1.2 point increase, p=0.375	0.6 point decrease, p=0.046	Non-specified	Non-specified	Regression of macular edema noted, stabilization but no regression in neovascularization	Non-specified	No significant differences
Noe A, et al. [9]	−0.2 mg/L, −0.5 mg/L, −1.5 mg/L, and −1.7 mg/L with the 0.1-, 0.3-, 1.5-, and 10-mg/kg doses, respectively; all p < 0.05	Non-specified	reduction of 0.31% at week 12 with 10 mg/kg (P = 0.038), reduction of 0.23% at week 4 with 1.5 mg/kg (P = 0.011)	Non-specified	Non-specified	Non-specified	Non-specified	No significant differences
Howard C, et al. [18]	Non-specified	Non-specified	Non-specified	Non-specified	Non-specified	Non-specified	Non-specified	No significant differences
Hensen J, et al. [19]	Non-specified	Non-specified	-0.18% difference vs placebo; multiplicity-adjusted, P=0.13902 with 50mg dose	No significant difference	No significant difference in HOMA-insulin resistance	Non-specified	Non-specified	No significant differences
Ridker PM, et al. [20]	36.4%, 53.0%, 64.6%, and 58.7% reduction for the 5-, 15-, 50-, and 150-mg doses, respectively, all p ≤0.02	23.9%, 32.5%, 47.9%, and 44.5% reduction respectively, all p ≤ 0.008	Modest but non-significant reduction	Modest but non-significant reduction	Modest but non-significant reduction	Non-specified	Non-specified	No significant differences
Rissanen A, et al. [21]	Non-specified	Non-specified	Non-specified	Favourable but non-significant reduction	Increase of 3.81 point estimate, p = 0.0525; increase in peak insulin level and insulin AUC also statistically significant	Non-specified	Non-specified	No significant differences

Risk of Bias

**Figure 2:**
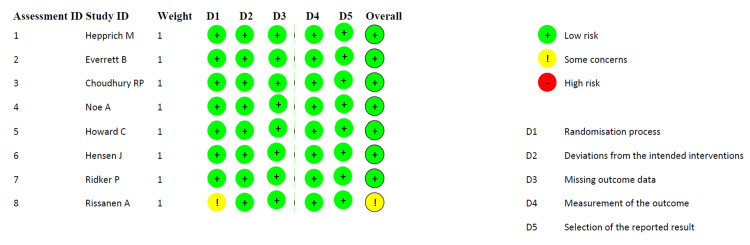
Risk of Bias across each domain for individual studies as per the Cochrane Risk of Bias Assessment Tool for Randomized Clinical Trials (RCTs) Figure generated using RoB 2 tool [24].

**Figure 3:**
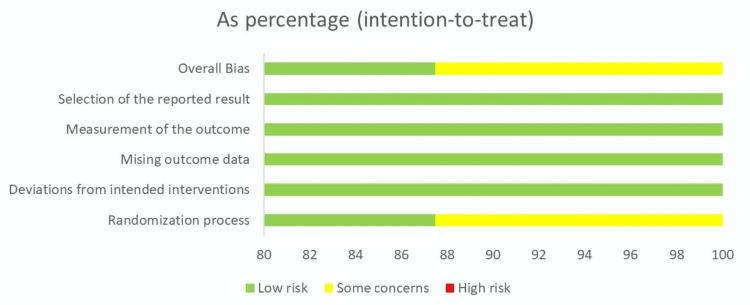
Overall Risk of Bias for all included Randomized Clinical Trials (RCTs) Figure generated using RoB 2 tool [24].

Appropriate quality assessment tools were used to evaluate the risk of bias in each study. The Cochrane Risk of Bias Assessment Tool was employed for RCTs which evaluates each study across five domains - randomization process, deviation from intended intervention, missing outcome data, measurement of outcome and selection of the reported data [22]. The CONSORT checklist extension for pilot and feasibility studies was used for the pilot study [23]. All studies were found to be low-risk except one by Rissanen et al. which had some concerns in the randomization process and selection of the reported result. All eight RCTs and the pilot study were included in the final review. A pictorial representation of the risk of bias calculated in each domain for all studies is included below in Figure 2. A tabular representation of the overall risk of bias in the studies assessed, which was found to be low, is also included in Figure 3 below.

Results of Individual Studies

Everett et al. tested the hypothesis that canakinumab reduces incident T2DM. They randomized 10,061 patients with prior MI and hsCRP≥2 mg/l to placebo or canakinumab at doses of 50 mg, 150 mg or 300 mg subcutaneously once every three months. During the median follow-up period of 3.7 years, they found that treatment with canakinumab did not reduce the incidence of new-onset T2DM with rates per 100 person-years in the placebo, 50 mg, 150 mg, and 300 mg canakinumab groups of 4.2, 4.2, 4.4, and 4.1, respectively (log-rank p=0.84). Additionally, although there was a reduction in HbA1C in the six to nine month period, there were no consistent long-term effects on HbA1C or BSL. However, canakinumab did have a significant effect on MACE in both those with and without T2DM (HR: 0.85; 95% CI: 0.70 to 1.03), pre-diabetes (HR: 0.86; 95% CI: 0.70 to 1.06), and normoglycemia (HR: 0.81; 95% CI: 0.49 to 1.35). Another noteworthy finding was that increasing tertiles of hsCRP at baseline were associated with an increased risk of developing diabetes (incidence rates 3.2, 4.1, and 4.4 per 100 person-years; p=0.003) [8]. 

Ridker et al. conducted an RCT of 556 patients with T2DM and high cardiovascular risk divided into 5, 15, 50, 150 mg canakinumab or placebo monthly groups. At a follow-up period of four months, it was found that canakinumab had a modest but non-significant effect on HbA1C, BSL and insulin levels. No effect was seen on low-density lipoprotein (LDL), high-density lipoprotein (HDL) and non-HDL cholesterol while triglyceride (TG) levels increased ≈10% in the 50 mg (p=0.02) and 150 mg (p=0.03) groups. In contrast, significant reductions were noted in hsCRP (36.4%, 53.0%, 64.6%, and 58.7% for the 5, 15, 50, and 150 mg doses, respectively vs 4.7% for placebo, all p ≤0.02); IL-6 (23.9%, 32.5%, 47.9%, and 44.5%, respectively vs 2.9% for placebo, all p≤0.008), and fibrinogen (4.9%, 11.7%, 18.5%, and 14.8%, respectively vs 0.4% for placebo, all p≤0.0001) [20].

Choudhury et al. sought to study the effect of canakinumab on arterial structure and function in 189 patients with atherosclerosis and T2DM/IGT via MRI. At a follow-up period of 12 months, they found that there was no statistically significant change in mean carotid wall area and no effect on aortic distensibility (change in mean carotid artery wall area was -3.37 mm2 with canakinumab vs placebo). However, there were significant reductions noted in hsCRP (GMR: 0.56; 95% CI: 0.414 to 0.758; p=0.0002) and lipoprotein(a) levels (-4.30 mg/dl (range: -8.5 to -0.55 mg/dl); p=0.025) while there was no statistical difference in OGTT levels and an increase in TG (GMR: 1.20; 95% CI: 1.046 to 1.380; p=0.01) [16].

Noe et al. conducted a dose-escalation study to evaluate the pharmacokinetic and pharmacodynamic characteristics of single-dose canakinumab in patients with T2DM. Two hundred thirty-one patients were randomly assigned to receive a single intravenous dose of canakinumab 0.03, 0.1, 0.3, 1.5, or 10 mg/kg or placebo with changes in hsCRP and HbA1C levels assessed at weeks four, eight, 12, and 24. It was found that dose-related reductions in hsCRP were significantly greater with canakinumab compared with those with placebo at week four (-0.2 mg/L, -0.5 mg/L, -1.5 mg/L, and -1.7 mg/L with the 0.1, 0.3, 1.5, and 10 mg/kg doses, respectively; all p<0.05) with significant reductions in hsCRP maintained up to week 12 with the two highest doses of canakinumab (-0.8 mg/L with 1.5 mg/kg and -1.3 mg/L with 10 mg/kg; both, p<0.05). A placebo-adjusted decrease in HbA1C was also found - 0.31% at week 12 with canakinumab 10 mg/kg (p=0.038) and 0.23% at week four with canakinumab 1.5 mg/kg (p=0.011) [9].

Howard et al. performed a pooled analysis of three RCTs in 1026 T2DM patients to study the safety and tolerability of canakinumab. Canakinumab groups were categorized as low (0.03 mg/kg iv once; N=20), intermediate (0.1 and 0.3 mg/kg iv once, 5 and 15 mg sc monthly; N=247), medium (1.5 mg/kg iv once, 50 mg sc monthly and 150 mg sc once; N=268), and high doses (10 mg/kg iv once and 150 mg sc monthly; N=137) and compared with placebo. An increase in average total cholesterol and TG levels was seen in patients treated with medium and/or high doses of canakinumab for at least the initial four months. Average HDL-C was higher in the high-dose and medium-dose groups during the first two months of treatment. Overall, the study found that canakinumab was safe and well tolerated over a treatment period of up to 1.4 years at the four pooled doses evaluated [18].

Hensen et al. randomized 551 patients to receive 5, 15, 50, 150 mg of canakinumab or placebo monthly as add-on to metformin therapy over four months. They found that all doses numerically lowered HbA1C from baseline between 0.19% and 0.31% (placebo-unadjusted), with maximal effect noted in the 50 mg dose of canakinumab (-0.18% difference vs placebo; multiplicity-adjusted, p=0.13902) while all other glycemic control parameters (FPG, fasting insulin, plasma glucose AUC(0-4h), 2-h PPG, peak glucose, C-peptide AUC(0-4h), peak C-peptide, insulin AUC(0-4h), peak insulin, ISR(0-2h), HOMA-β and HOMA-IR) remained unchanged [19].

Rissanen et al. aimed to study the effect of canakinumab on insulin secretion and beta-cell function by randomizing 190 patients with T2DM or IGT to receive canakinumab vs placebo. At four weeks, ISR relative to glucose at 0-0.5 h numerically favored canakinumab vs placebo in insulin-treated patients (PE 3.81 pmol/min/m(2)/mmol/l; p=0.0525) and in the IGT group (PE 3.92 pmol/min/m(2)/mmol/l; p=0.1729) while ISR relative to glucose at 0 to two hours or other time points were not statistically significant for canakinumab vs placebo across groups. Mean change from baseline in fasting plasma glucose favored canakinumab in the metformin group and the IGT group; however, differences were not statistically significant. Mean change from baseline in peak insulin level and insulin AUC 0 to four h were statistically significantly higher in the canakinumab group in IGT patients [21].

Hepprich et al. conducted an RCT to assess the efficacy of canakinumab plus standard-of-care compared with placebo plus standard-of-care in 116 patients with T2DM and a BMI>25 kg/m^2^ hospitalized with SARS-CoV2 infection in seven tertiary-hospitals in Switzerland. There was no statistically significant difference in HbA1C after four weeks despite a lower number of anti-diabetes drugs administered in patients treated with canakinumab. Meanwhile, hsCRP and IL-6 were found to be lowered by canakinumab [15].

Stahel et al. conducted a prospective pilot study to evaluate the effect of systemic interleukin-1β inhibition on retinal neovascularization in proliferative diabetic retinopathy. Six patients were given canakinumab 150 mg sc three times and the follow-up period was 24 weeks. A not statistically significant reduction in retinal edema was detectable while the area of neovascularization and best-corrected visual acuity remained unchanged. Additionally, mean HbA1C improved significantly from 7.92% to 7.30% (p=0.046) however systemic inflammatory parameters remained overall unchanged [17].

Discussion

Our systematic review found that overall, canakinumab had a modest but mostly non-significant effect on glycemic parameters including HbA1C and BSL. Three out of nine studies found canakinumab to cause a significant reduction in HbA1C. However, of these, Everett et al. found a reduction in HbA1C at six to nine months but noted no consistent long-term effects and while Stahel et al. found a significant reduction in HbA1C at a follow-up period of 24 weeks, the study design of a prospective pilot study, limited sample size of six patients and lack of a control limit the validity and reproducibility of these findings [8,17]. Noe et al. found a placebo-adjusted decrease in HbA1C at week 12 with the 10 mg/kg dose and at week four with the 1.5 mg/kg dose [9]. To summarize, canakinumab was observed to cause an early, transient fall in HbA1C levels, especially with higher doses but this effect is not sustained long-term or statistically significant in most studies.

On the other hand, canakinumab was found to cause a consistently significant reduction in systemic inflammatory parameters like hsCRP and IL-6. Interestingly, increasing tertiles of hsCRP at baseline were found to be associated with an increased risk of developing T2DM by Everett et al. [8]. In terms of diabetic complications, canakinumab was found to have positive effects on the incidence of MACE. While this is consistent with previously published data, it is important to note that canakinumab has been found to reduce MACE in both patient populations with and without T2DM. It was also found to have a moderate yet not statistically significant reduction in diabetic retinal edema.

In addition, canakinumab was found to be associated with some increase in TG levels but was overall deemed to be safe and tolerable in all patient populations.

Our research suggests that while canakinumab significantly reduces markers of subclinical inflammation like hsCRP and IL-6 (which do predict development of T2DM) and incidence of MACE in patients with overt diabetes, pre-diabetes and normoglycemia; it does not reduce rates of incident T2DM or cause a significant sustained reduction in HbA1C levels in those with T2DM or IGT. We find that our data is agreeable with existing literature on the topic [8,9,25]. While inflammation does seem to play a significant role in both T2DM and atherosclerotic cardiovascular disease in accordance with the ‘common soil’ hypothesis, there seems to be a divergence in the inflammatory pathways implicated in both entities at some point, as suggested by our own data and underscored by the CANTOS trial [11].

However, just as alternative anti-inflammatory agents failed to show clinically relevant effects on MACE prior to CANTOS, a search for alternative drugs targeting various other inflammatory pathways is reasonable and may yet yield significant results in the arena of diabetes prevention and control [26,27]. We must consider that beyond IL-1, other cytokines may drive the progression from pre-diabetes to T2DM. For instance, adipose tissue can produce tumor necrosis factor, a cytokine implicated in insulin resistance in mouse models of T2DM and drugs targeting this pathway warrant further investigation in well-designed studies [28]. Another example is salicylate, an anti-inflammatory agent with less specific targeting, which is believed to inhibit nuclear factor kappa-β (NF-Kβ) activity by activating adenosine monophosphate-activated protein kinase, causing a modest reduction in HbA1C and inflammatory markers in patients with T2DM [29]. Additionally, the ongoing Cardiovascular Inflammation Reduction Trial (CIRT) of low-dose methotrexate in patients with established coronary artery disease and diabetes or metabolic syndrome is monitoring incident T2DM [30]. Thus, our ongoing quest for elucidating and targeting clinically appropriate inflammatory pathways for a landmark advancement in the control and prevention of T2DM is not only warranted, but also necessary in the purview of increasing diabetes-associated global morbidity and mortality.

As with any systematic review, this study too had its inherent limitations. Firstly, the search strategy was limited to three databases and considered only free full texts in the English language, leading to a possible language bias and to missing out on potentially relevant studies. Secondly, one cannot disregard the inherent publication bias especially with the inclusion of retrospective studies and the limited generalizability in view of the inclusion criteria. In addition, the inclusion of a prospective pilot study with a limited sample size of six patients and lack of a control leads to a possible selection bias. Thus, future studies are warranted to fill these potential gaps.

## Conclusions

We found that canakinumab had a modest but mostly non-significant effect on glycemic parameters including HbA1C and BSL, with an early transient fall in HbA1C with larger doses but no significant sustained reduction. It did cause a consistently significant reduction in systemic inflammatory parameters like hsCRP and IL-6, with increasing tertiles of hsCRP also found to be associated with increased rates of incident T2DM. Canakinumab was found to reduce rates of MACE in patients with overt T2DM as well as in populations with pre-diabetes and normoglycemia as well as cause a not statistically significant reduction in retinal edema. In addition, it was found to be safe and well-tolerated in all patient populations.

While inflammation does seem to play a significant role in both T2DM and atherosclerotic cardiovascular disease in accordance with the ‘common soil’ hypothesis, there seems to be a divergence in the inflammatory pathways implicated in both entities at some point, as suggested by our own data and underscored by the CANTOS trial. Thus, exploration of alternative inflammatory pathways and targets for the prevention and control of T2DM is warranted.
